# Application of Incident Command Structure to clinical trial management in the academic setting: principles and lessons learned

**DOI:** 10.1186/s13063-016-1755-9

**Published:** 2017-02-09

**Authors:** Penny S. Reynolds, Mary J. Michael, Bruce D. Spiess

**Affiliations:** 1 Department of Anesthesiology, Virginia Commonwealth University Medical Center, Richmond, VA USA; 2 Present address: Department of Anesthesiology, University of Florida, Gainesville, FL USA

**Keywords:** Incident Command Structure, ICS, Trial failure, Trial management

## Abstract

**Background:**

Clinical trial success depends on appropriate management, but practical guidance to trial organisation and planning is lacking. The Incident Command System (ICS) is the ‘gold standard’ management system developed for managing diverse operations in major incident and public health arenas. It enables effective and flexible management through integration of personnel, procedures, resources, and communications within a common hierarchical organisational structure. Conventional ICS organisation consists of five function modules: Command, Planning, Operations, Logistics, and Finance/Administration. Large clinical trials will require a separate Regulatory Administrative arm, and an Information arm, consisting of dedicated data management and information technology staff. We applied ICS principles to organisation and management of the Prehospital Use of Plasma in Traumatic Haemorrhage (PUPTH) trial. This trial was a multidepartmental, multiagency, randomised clinical trial investigating prehospital administration of thawed plasma on mortality and coagulation response in severely injured trauma patients.

We describe the ICS system as it would apply to large clinical trials in general, and the benefits, barriers, and lessons learned in utilising ICS principles to reorganise and coordinate the PUPTH trial.

**Results:**

Without a formal trial management structure, early stages of the trial were characterised by inertia and organisational confusion. Implementing ICS improved organisation, coordination, and communication between multiple agencies and service groups, and greatly streamlined regulatory compliance administration. However, unfamiliarity of clinicians with ICS culture, conflicting resource allocation priorities, and communication bottlenecks were significant barriers.

**Conclusions:**

ICS is a flexible and powerful organisational tool for managing large complex clinical trials. However, for successful implementation the cultural, psychological, and social environment of trial participants must be accounted for, and personnel need to be educated in the basics of ICS.

**Trial registration:**

ClinicalTrials.gov, NCT02303964. Registered on 28 November 2014.

## Background

Clinical trials are essential to evidence-based medicine, but failure rates can be high [1, 2]. Failed trials result in both substantial cost burdens and risk to patients without evidence of benefit. Lack of trial success may result from product failure (that is, inability of the test intervention to demonstrate either clinical benefit or an acceptable safety profile), but poor management may also be a contributing factor. Although hard data on the specifics of trial management failures are difficult to come by [3, 4], there is no reason to believe that clinical trials are exempt from the management problems found in other sectors. For example, it has been estimated that more than 75% of information technology (IT) projects fail, with over 30% cancelled before completion, and more than 50% with significant cost over-runs. Management inadequacies account for the bulk of failures. Specific management problems include inadequate project organisation and planning, inadequate leadership and governance, overly optimistic projections of project feasibility, inappropriate and inadequate research team skills and competency, and ineffective communication between invested parties [5].

**Fig. 1:**
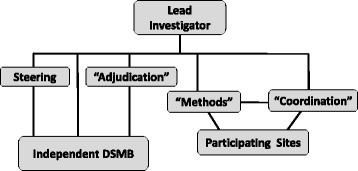
Conventional clinical trial management architecture (adapted from [9]). This is ‘flat land’ management [10], as the lead investigator is directly involved with all aspects of trial management. Although communication chains are short, the lead investigator can be easily overwhelmed by information and low-level decision-making

Effective management of all features of a trial should increase the chances of successful trial completion [6, 7]. However, although there is extensive documentation on regulatory aspects of running clinical trials [4, 8], few practical guidelines deal explicitly with the project management side [7]. Commonly, trials are organised on a committee basis (Fig. 1). In this model, team units consist of a *steering committee*, involving the lead or principal investigator (PI) and other coinvestigators who share the overall responsibility for the trial; a *safety adjudication* committee of physicians to review safety and protocol compliance; an independent *Data Safety Monitoring Board* (DSMB); and a *methods* committee, which has oversight for day-to-day operations. Personnel consist of physicians, research coordinators, research nurses, administrators, trial-specific staff, and one or more biostatisticians [9]. Because the lead investigator is directly involved with all aspects of trial operations with few or no intermediate levels of organisation, this is essentially a ‘flat land’ management structure [10].

Flat land management has significant disadvantages. As the legally responsible entity for the trial, the lead investigator is implicitly assumed to understand and be involved with most, if not all, aspects of trial management. These include a diverse array of tasks, including scientific and grant development, trial planning, budget and resource allocation, trial design, trial logistics, project and personnel management, and accountability for all required regulatory mandates [11]. However, academic lead investigators must juggle many other responsibilities related to their primary job [12, 13], and may get little protected time away from clinical responsibilities. Many tasks, especially those pertaining to budgeting and regulatory compliance, are highly complex and may be nearly invisible to the lead investigator, who will not have the necessary training, expertise, or time for adequate oversight. As a result, most trial-related activities are delegated to a trial coordinator (TC), with the expectation that this person will somehow ‘make it all work’. Proficiency requirements for certified Clinical Research Coordinators (CCRCs) now include over 100 tasks in five domains (investigational product management, protocol development, safety, regulatory issues, and trial management) and approximately 20 subject areas [14]. Nevertheless, although these individuals might have the expertise to direct the overall operational aspects of a clinical trial, they cannot be expected to have the additional scientific background, financial and business acumen, and in-depth knowledge required to manage all tasks required for a particular study. In a large trial, leadership can be easily overwhelmed by the amount of unfiltered information to be processed and the number of low-level decisions to be made [10]. Consequences include unrealistic expectations as to what the trial can accomplish, ineffective planning, poor integration of evolving trial requirements into the management structure, poor decision-making, poor communication, inefficient delegation of responsibilities and workloads, lack of accountability, confusion, and squandered personnel and financial resources.

The ‘gold standard’ for managing complex operations in the major incident and public health arenas is the Incident Command System (ICS). We used it to reorganise and manage a large, multigency, multidepartmental clinical trial, the Prehospital Use of Plasma in Traumatic Haemorrhage (PUPTH) trial [15]. This article describes the fundamentals of the ICS, and our experience and lessons learned in implementing this model to coordinate the diverse agencies, clinical departments, personnel, and tasks required to launch the PUPTH trial.

## Methods

The Incident Command System (ICS) is a systematic tool used for the command, control, and coordination of emergency response-requiring direction and synchronisation among several groups, agencies, or organisations. It is a hierarchical organisational structure that clearly depicts lines of authority, functional roles and responsibilities, and communication networks [16]. ICS was first developed in the 1970s by wildland fire services in California and Arizona, and has since been modified and adapted for use during local incidents such as major traffic accidents [17], hospital surge response [18], planned mass events (such as the Vancouver 2010 Olympics [19]), and epidemic disease outbreaks [20–22].

ICS is characterised by modular organisation and coordination of functional groups, rather than direct management by the lead investigator of individual assets or personnel. ICS modular organisation determines a manageable span of control that results in increased efficiency, flexibility, and above all, accountability. In the early stages of project planning, or for very small trials, initial management of the most important activities can be the responsibility of a few people. As the trial increases in size and complexity, personnel can be assigned to functional units as needed, and according to their expertise and skillsets. Roles can be discontinued when no longer required, and personnel reassigned to different positions (‘role switching’) within the trial [23]. Each supervisory level is managed by a *liaison,* who reports directly to the designated project leader and is responsible for managing the appropriate number of tasks and personnel compatible with maintaining effective oversight and communication. Teams are small, cross-functional, self-directed, and able to establish team goals and assess progress. The lead investigator, although ultimately responsible for all aspects of trial conduct, takes only those actions that cannot be made by liaisons reporting directly to the project lead. As a result, the organisational structure is never larger than required, but is sufficient to maintain a reasonable span of control and efficiency, and accomplish the required tasks without spreading personnel too thin.

**Fig. 2:**
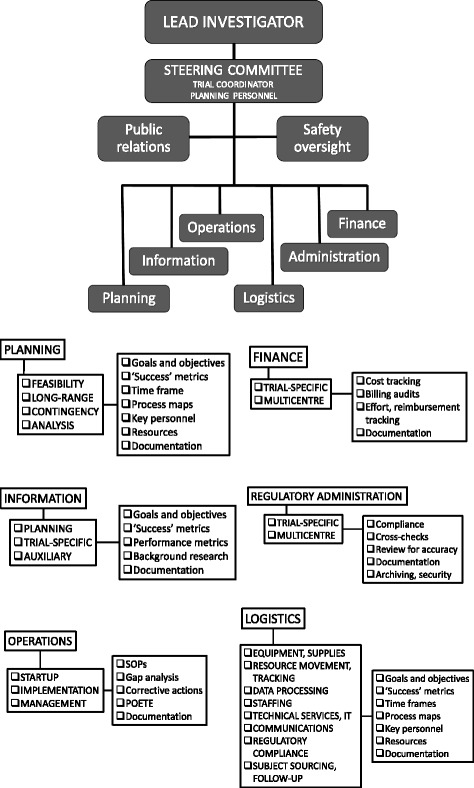
Incident Command Structure as applied to clinical trial management. Advantages are more manageable span of control, more effective coordination of diverse tasks, and more effective allocation of tasks and functions to teams with specific expertise and oversight skills

The standard ICS model consists of five functional modules: *Command*, *Planning*, *Operations*, *Logistics*, and *Finance Administration* [24]. To streamline clinical trial organisation, the basic ICS structure can be modified to include additional *Regulatory Administration* and *Information* arms (Fig. 2).

## Command

Command staff includes the *lead/principal investigator*, *trial coordinator/project manager*, *steering committee*, *safety oversight officer*, and *public relations officer.*

The *lead investigator* is legally responsible for trial oversight. For larger trials, general management tasks (clinical research coordination and trial management, safety, and public relations) are delegated to Command staff, which includes the TC and one or more assistants. However, effective oversight and involvement of the lead investigator and TCs cannot be achieved by delegation alone unless adequate oversight and monitoring mechanisms are in place [25, 26]. These mechanisms involve the provision of regularly updated and consistent information on trial operational status. To prevent the lead investigator and TC from being overwhelmed by detail, management staff must function as a united team, coordinating plans and actions across the trial, and exchanging timely, accurate, and consistent information with all study personnel. Delegation of authority, and roles and responsibilities of all key personnel must be clearly defined. To maintain continuity and ensure legal compliance, alternates must be designated in case of prolonged absence, illness, or turnover of key personnel. For industry-sponsored trials, study oversight is provided by an independent academic *steering committee*. The steering committee is composed of academic investigators, and should have authority over critical decisions related to trial conduct, data collection and management, reporting of results, and safety monitoring and oversight. An outside biostatistician should have independent access to the raw data and be able to conduct data analyses independent of the sponsor [27].

*Safety* oversight is usually provided by one or more physicians responsible for monitoring subject accrual, compliance with enrolment and eligibility criteria, and adverse events. The trial safety officer liaises directly with the Data Safety Monitoring Board (DSMB), the members of which are not associated with the trial, and work independently of the lead investigator. The purpose of the DSMB is to provide arm’s-length independent oversight of the safety of trial patients, and to monitor overall trial conduct; at least one member should be an independent biostatistician [28]. Formalised standardised templates for DSMB management and responsibilities are available (e.g. https://humansubjects.nih.gov/data_safety; [29].

*Public Relations and Communications* involve the development, integration, and management of effective communication between the research entities and the public. Effective public relations (PR) are essential to the recruitment and enrolment effort for clinical trials. They also fulfil an important informational role in public education as to the purpose and benefits of the proposed trial. In the academic setting this responsibility is generally undertaken by an institutional office of communications and public relations. However, a study-specific strategic communications plan may be required, especially if the research is controversial or subject to misperception. Community notification and consultation meetings will be required when research is conducted under Exception to Informed Consent. A PR officer may be designated for communicating with the institutional PR officer and other trial stakeholders. An information dissemination plan should be put in place early in the trial so that essential activities can be identified and budgeted; these include crisis management, community notification meetings, and initiation and updating of relevant web sites, including clinical trial registration [30].

## Liaisons

*Liaisons* interact between Command staff and other operational groups. Each liaison is responsible for a specific operational group, which in turn manages specific assignments. Liaisons should have the autonomy to select team members to achieve the tasks delegated to that functional group. They are, therefore, accountable for completion of those tasks, and providing group activity reports required for regulatory documentation; these activity reports are also means of tracking personnel effort for fiscal accounting and project reimbursement. Expectations for each liaison and operational group must be clearly communicated, and strategies for achieving liaison buy-in and accountability must be in place.

## Planning

Planning is the process of defining strategies, strategic priorities, major tactics, and the coordination of all support activities to accomplish the objectives of the clinical trial. The Planning section supports the Command and Operations sections through the processing of information relevant to trial status, developing and updating action plans, providing recommendations and directives, and supplying all pertinent documentation. In addition to planning of the trial proper, planning activities will also include pretrial *feasibility planning, long-range planning*, *contingency planning*, and *analysis planning*.

*Feasibility planning* should be a realistic assessment of the capacity of the research team to successfully execute the proposed trial. It should be the first step in trial planning, and should be completed well before the proposed trial initiation date [31]. Almost one third of trials fail because no subjects could be enrolled, and 80 to 90% of trials do not meet enrolment timelines [12]. Thus, the feasibility plan should provide verifiable and accurate forecasts of subject availability and recruitment rates, preferably based on relevant historical or epidemiological site data. Other measures of feasibility include success of prior trials and trial partnerships; realistic assessment of personnel, staffing, and resource capacity; and an estimate of likely budget requirements [32, 33].

*Long-range planning* forecasts anticipated resource needs (e.g. supplies, personnel, staffing) and subject recruitment projections. Identification of specific needs requires close collaboration with, and feedback from, each of the relevant service groups involved in the trial.

*Contingency plans* should be developed to anticipate unexpected and disruptive changes in the trial and identify alternative courses of action. In any trial the chances of something going wrong is virtually 100%. Contingency planning involves the identification of potential risks for each functional area and the negative consequences of each, prioritisation of those risks, and development of backup plans to minimise disruption of the trial should one or more of these events occur. Likely risks include lower than expected subject recruitment rates, lower than expected event rates, significant adverse events, loss or absence of key personnel, data loss, and data security breaches.

*Analysis planning* includes issues pertaining to study experimental design (including randomisation and blinding), study sample size estimation, choice of statistical models, interim analyses [33], methods used to estimate treatment effects, and criteria determining clinical importance (as opposed to statistical significance) [34]. It will require the participation of one or more professional statisticians.

All stakeholder personnel (including service groups) should be regarded as active collaborators with a vested interest in the research, rather than mere consultants (or, as in one investigator’s appalling phrase, ‘blue-collar workers’). Detailed planning and specific tactics will be determined by each of the relevant service groups involved in the trial. Personnel investment in the trial (‘buy-in’) is improved and maintained by clear communication of expectations, regular updates of trial status, determining the appropriate skillsets required for specific jobs, provision of timely and adequate support so that personnel can do their job, and showing active appreciation and reward. Expectations for coauthorship and financial remuneration must be clarified and discussed early.

To ensure agreement and common purpose in all members of the planning team and to avoid mission creep, the objectives, purpose, and scope of the trial should be clearly identified at the very beginning. The primary and secondary outcome variables to be measured (*what*) are identified, prioritised, and endorsed by the team. After that, the *how* and *who* need to be identified: the methods and resources required to collect, disseminate, analyse, and secure relevant information, and the specific people identified with the requisite skills who can do the work. Open communication between all stakeholders is a priority, especially with key service groups responsible for data collection and management. Information must be timely, accurate, and useful, otherwise personnel are forced to work in a vacuum. As a result, the planning committee should include representatives from all key functional areas and technical specialties, and not be restricted to coinvestigators and physicians, as is generally the case.

### The planning process

The common elements to the overall trial plan include a clear statement of *goals and objectives*, clearly defined *success metrics* (how will you know the intervention or process ‘works’?), a defined *time frame* and time periods for all operations, *process and procedure flow maps*, identification of *key personnel* to be in charge and the reporting process to be followed, a preliminary *resource list* (to be further refined by input from liaisons for each functional area and service group), and methods to *document* the planning process. Identification and correction of operational inefficiencies or gaps must be performed in collaboration with other functional groups, especially Operations and Logistics. A clear statement of goals and objectives is necessary to ensure agreement and common purpose in all members of the planning team and to avoid mission creep. The primary and secondary outcome variables to be measured (*what*) are identified and prioritised. After that, the *how* and *who* need to be identified: the methods and resources required to collect, disseminate, analyse, and secure relevant information, and the specific people with the requisite skills who can do the work.

**Fig. 3:**
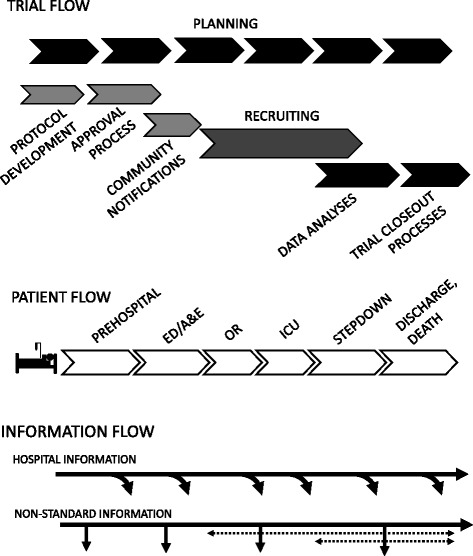
Generalised task flow maps for the PUPTH trial. Integration of Patient Flow with Information Flow maps was crucial for identifying barriers to data collection; blood samples (*non-standard information*) had to be collected at predesignated time points; however, patient location in the hospital care chain at those times could not be predicted in advance

The plan requires *process flow maps.* These maps are a schematic timeline of each of the required tasks or activities in the order in which they need to be accomplished (Fig. 3). The sequence shows both *task dependencies* (what tasks need to be accomplished before another given task), and *functional dependencies* (when tasks or processes cross functional boundaries, such as a different database or service department required to obtain or process data) [35]. Process mapping facilitates the identification of personnel groups required to perform a given activity, immediate needs (including missing or redundant tasks), points of workflow interactions, points of documentation, permissions to be obtained, and databases utilised. The flow map can be supplemented as needed with estimates of time taken to complete each step, resources, and costs [36, 37]. Several task maps may be required; for example, a general trial flow map, a map of patient or subject flow through the system, and critical data acquisition and collection points (Fig. 3). Additional process maps can be created for the oversight review and approval process, contracts and budgeting, and final preparation (e.g. site preparation, sponsor site visits, study drug or device acquisition and shipping) [35].

Because plans are to be communicated to everyone involved in the trial, they should be simple, concise, and clearly written. The amount of detail in the general plan should be sufficient for a clear understanding of trial goals and overall feasibility. All personnel must be informed as to their roles and responsibilities related to a plan, and provision made for the necessary training. Whenever practical, ‘boots on the ground’ rehearsals or drills should be conducted to identify weaknesses and problems, and begin corrective action. Plans must be reviewed on a regular basis, especially if there are major changes in personnel, operations, and technology. Specific milestones or targets must be set to measure progress, identify specific personnel designated with the responsibility for accomplishing each task, and an established method for reporting established. People with both leadership authority and a genuine interest in having the project succeed should be assigned to mission-critical tasks.

## Information

The true deliverable of a clinical trial is information that is accurate, reliable, and secure. Therefore, it is essential that a dedicated data management and IT team is involved in the early stages of clinical trial planning, both to avoid mission creep and ensure appropriate alignment of data collection requirements with study objectives. The activities involved in information management are diverse, consisting of variable identification and coding, data collection, data collation, matching of diverse databases, transfer, archiving, analyses, documentation, access, quality control, and security. Permissions and handling of sensitive information are integral to the process. Biostatisticians will need to collaborate with IT and data management groups to identify and work through data management issues. Sufficient planning time must be allocated to determine the most appropriate experimental design, sample sizes, and analytical methods for the study. These require an adequate understanding by all parties of the scientific question, and the form and origin of the data. Extraneous or ‘non-goal’ trial data (data not associated with primary or key secondary endpoints, regulatory compliance, or standard baseline assessments) result from a loss of focus, and contribute to increased trial costs, while diluting trial effectiveness [38].

There are three main information streams: *trial-specific information*, *planning information*, and *auxiliary information. Trial-specific information* consists of data on patient demographics and primary and secondary outcomes. Data are obtained from standard medical records (as part of the documentation of patient care), and/or from non-standard sources. Non-standard data are required meet the objectives of the study, but are not part of routine medical care (for example, blood samples used to identify novel biomarkers). *Planning information* is used for internal planning and contingency discussions designed to address trends, associations, problems, milestone assessments, or other issues indicated by the metrics, and enables appropriate corrective action to be made in a timely way. Planning information relies on the collection of benchmark metrics – easily measured, readily available, relevant data related to study implementation and trial conduct (rather than patient outcomes). Predetermined and clearly defined metrics permit major decisions to be made with reference to hard data, rather than by intuition, prior experience, or HiPPO (Highest Paid Person’s Opinion) syndrome [39]^1^. Performance metrics include time taken to complete the critical steps of all major processes or tasks, number of errors (such as missing data, out of bounds data entries, protocol deviations), patient enrolment numbers, number of eligible patients, number of missed enrolments, protocol deviations, expenditures and costs, and capacity gaps (resources required versus resources supplied).

*Auxiliary information* relates to all background and supplementary information pertaining to the trial. Both systematic and rapid reviews are increasingly recognised as an essential part of the planning process for new clinical trials [40, 41]. A synthesis of all relevant evidence is necessary to ensure that the proposed research avoids unnecessary duplication, addresses relevant deficiencies in previous work, and minimises research waste [42]. Professional librarians are information specialists, and can assist with literature searching, citation management, and evidence appraisal.

### Operations

The goal of appropriate operational management is to make the trial ‘work’; that is, to fulfil the strategic goals and objectives of the trial. Operations consist of all the tasks and resources required for trial start-up, implementation, monitoring, and management. These are facilitated by development of written standard operating procedures (SOPs).

SOPs are brief (one to two-page) written guidelines that define how operations are to be conducted, and expectations and requirements for all personnel when performing their job [43, 44]. Personnel working in different departments or functional groups often work in virtual silos and are unfamiliar with the requirements, organisation, and functional response of other groups. SOPs are, therefore, indispensable for familiarising personnel with trial procedures, ensuring compliance with regulatory requirements, minimising protocol deviations or violations, information sharing and coordinating effort across the trial, and identifying structural barriers that result from working across administrative divisions. SOPs that are applicable to institutional procedures may already be in place. In the US, the Food and Drug Administration (FDA) mandates documented SOPs specifically for data collection and handling in FDA-oversight trials, and these SOPs are subject to federal audit.

SOPs should be uniformly structured and consist of a formal statement of objectives, list of responsible personnel, a line-item list of procedures, and a list of definitions for all major regulatory terms and acronyms used [44–46]. Relevant guidelines and regulations should be referenced as applicable (e.g. ICH E6 Good Clinical Practice, 21 CFR 50; Exception from Informed Consent Requirement, 21 CFR 50.24). SOPs should be signed and dated by the responsible agent for the functional group, administrator, or director. All SOPs should be reviewed at regular intervals, and revised as necessary to ensure applicability to the trial [44–46].

The liaisons for each functional or service group are responsible for assessing operational gaps, instituting appropriate corrective action, and reporting to the planning committee in a timely way. This information will also be required for regulatory documentation. Gaps can be assessed using a standardised methodology such as POETE: Planning, Organisation, Equipment, Training, Evaluation [46].

## Logistics

Logistics refer to the management of all support activities to maintain the operational function of the clinical trial: equipment and supplies, movement of resources and personnel, processing of data, subjects, and staff, and technical services. Other support activities may include communication capabilities with other functional areas, service groups and relevant personnel; resource tracking; and personnel accountability. Logistic liaisons will interact with the finance sector for preparation of service and equipment contracts, and with the administration sector to ensure that personnel have on file all required regulatory compliance documentation, training certifications, and licensure requirements. Logistics personnel will also be involved with practical aspects of subject source identification and availability, determination as to feasibility of a single-centre versus multicentre trial, between-centre liaison strategies, decisions on treatment and control interventions, and subject follow-up strategies [47].

IT logistics can be especially challenging. Before trial launch, comprehensive policies should be in place for identification of sensitive information and rules for data sharing. Software compatibility between functional groups, and personnel training, familiarity, and comfort with software tools must be considered. Protocols for software tool use should be established, and full use of tools rehearsed.

Frequently, grant applications are approved before all resources can be identified, and problems will become apparent only when the trial is close to, or even fully, operational. Thus, frequent status evaluations, updates, and process improvement implementation will be required, especially in the immediate implementation phase after trial start-up.

## Regulatory administration

Regulatory requirements contribute substantially to the costs and administrative burden of most trials [32]. Over the past few decades, protocols have become increasingly complex in both scope and oversight requirements, as the intent of the protocol has evolved from an overview of trial operations to a legal document requiring strict adherence. Consequently, staffing, staff responsibilities, and workload have also increased.

The success of a large clinical trial depends on organised, detail-oriented, experienced, and ethical research staff. The lead investigator must ensure that personnel are qualified by education, training, and experience (and licensure where relevant) to perform the delegated tasks. In general, the TC, a designated study nurse, or regulatory specialist, will be the primary manager of regulatory compliance tasks. However, two or more dedicated personnel may be required, depending on the amount and complexity of the workload and the size of the trial. Ensuring that staffing is sufficient for the workload is critical, as regulatory specialists may be responsible for anywhere up to six protocols at a time, all at different stages of management and implementation [32]. If the regulations are unclear or are questionable, the lead investigator and regulatory delegates are responsible for their clarification. All personnel involved in the trial must understand that they are responsible for communicating queries and areas of concern before taking action on their own. Lack of clarity is not synonymous with permission to make assumptions based on what is most convenient. The result of leaving actions to individual interpretation and assumption is non-compliance and a failure of the trial to protect and value research subjects.

## Finance

In the academic setting there is usually a specific administrative office that oversees the financial management of large grants. However, at least one dedicated person ‘on the ground’ should be employed to track costs, account for reimbursements, and perform billing audits. This is essential in cross-agency or cross-departmental studies involving separate administration of multiple subaccounts.

Cost over-runs and cash shortfalls are common in trial operations. Both sponsors and academic investigators frequently underestimate the true work effort, and therefore costs, involved in trial start-up and task performance. Sponsors often justify reimbursement rates in terms of industry benchmark services and procedure costs, and thus may significantly underestimate the actual personnel time and project costs in a non-industry setting. Procedural costs and billing may not be standardised across all centres or protocols in a multicentre trial. Various key personnel or groups may negotiate for additional payments out of the trial funding pool, without clear understanding of the overall budget allocations. If billing and payments are made on an installment basis, significant lags between payments may occur, making accounting difficult. Developing a reasonable reimbursement schedule requires as much specific information on time and effort as possible before the study is initiated. This will require collaboration with all functional groups and service areas, as information will be required for all line items and ancillary activities such as training time.

## Results

**Fig. 4:**
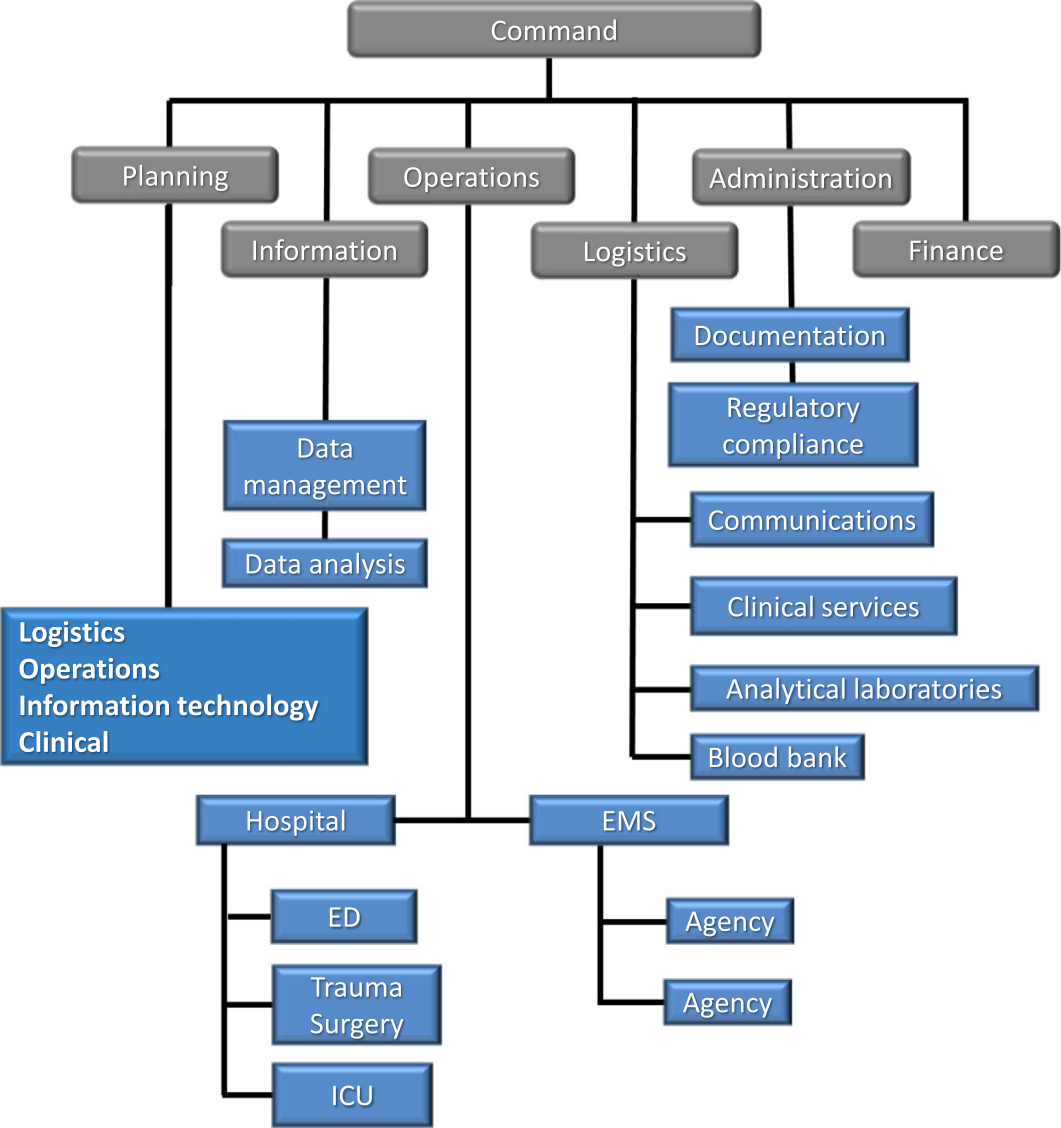
Incident Command Structure as applied to management of the PUPTH trial. This schematic clearly identified chains of command, responsible personnel, and task and service units. It made for more effective allocation and monitoring of tasks, simplified coordination and coverage of essential service personnel, and improved accountability

The Prehospital Use of Plasma in Traumatic Haemorrhage (PUPTH) trial was an investigator-initiated, prospective randomised clinical trial to determine the effect of prehospital administration of thawed blood plasma on mortality and coagulation response in severely injured trauma patients [15]. During the start-up phase of this trial, there was considerable pressure to get the trial launched quickly without ‘waste’ of time. Early planning was performed by trial coinvestigators, almost all of whom were clinicians. Without a management infrastructure or clear lines of accountability in place, planning tended to be haphazard, with no clear understanding of tasks or responsibility for task completion. Consequently, the first phase of the trial was characterised by confusion, delays and stagnation. In the second year the trial was reorganised along an ICS format (Fig. 4). The revised core management and planning team consisted of a dedicated TC, a logistics officer, an additional regulatory officer, and several experts in clinical data management and IT.

## Benefits

Comparison of the first and second years of the PUPTH trial made clear that ICS had several advantages:

1. The ICS organisational structure clearly identified chains of command, identified responsible personnel and task units, made for more effective allocation and monitoring of tasks, and improved accountability.
2. Targeting the expertise of relevant personnel in specific service groups improved management of limited resources and budget.
3. Task flow mapping facilitated coordination and coverage of essential service personnel (such as the transport and processing of blood samples).
4. ICS and sectional task flow mapping provided rapid identification and correction of areas of inertia, and waste, and allowed prompt identification of operational bottlenecks. Requests for corrective action could be targeted to the appropriate responsible parties.
5. ICS was particularly helpful for organising, coordinating, and streamlining the enormous workload involved with regulatory compliance. Instead of a single TC having the sole responsibility for meeting all compliance requirements, as is usual in clinical trials, the workload could be distributed more evenly over relevant personnel across the system.

## Barriers

The main barriers to effective ICS implementation were lack of acceptance of ICS-based organisation, lack of shared goals, priority conflicts, and communication bottlenecks:

1. Unfamiliarity with ICS culture. The PUPTH trial was the first time this research group formally implemented ICS in the management of a clinical trial. All personnel in both emergency medical service (EMS) agencies and several individuals on the planning team were familiar with the ICS system. However, unlike the fire-fighting and EMS agencies, clinical departments tend to be self-contained cultures, and physicians were unaccustomed to coordinating efforts across departments within a larger ICS structure. The lead investigator was clearly recognised as the final arbiter. However, clinical personnel unused to working within an ICS did not always recognise the authority of the TC and steering committee, and ignored routine communications. Clinicians often found it difficult to adjust to the concept that personnel in the ICS hierarchy were identified with specific roles to support trial organisational structure and needs, rather than by individual status or affiliation. Misunderstandings resulted from clinicians preferring to maintain existing comfort levels and ‘business as usual’. As a result, they either could not be relied on to fulfil designated responsibilities without oversight, or alternatively, took action on their own, circumventing procedures established to promote cross-organisational communication (‘free-lancing’ [23])
2. Lack of shared goals. A second barrier was the lack of a clearly defined ‘shared-value system’ that was understood and accepted by all trial participants [23]. The majority of individuals had the success of the trial as a priority, and were strongly motivated to make all aspects of the trial work. However, less-committed individuals or groups showed little initiative, required frequent follow-up and reminders to complete tasks, or perceived the role flexibility inherent to ICS organisation as a threat. This seriously impeded the day-to-day functioning of the trial
3. Priority conflicts. Demands of a clinical trial inevitably will be lower priority if they conflict with standard operations. Conflicts over resource and priority allocation resulted because of difficulties in meeting the demands of the trial simultaneously with the day-to-day operational requirements of the hospital and outside agencies. In the PUPTH trial, this was apparent in difficulties inherent in the management and distribution of thawed plasma, and resulting demands placed upon both prehospital personnel and the institutional blood bank
4. Communication bottlenecks were a persistent problem. Although the appropriate liaisons and alternatives had been designated early, key groups did not always notify the planning committee when personnel changes occurred. ‘Stove-piping’, poor motivation, and unexpressed lack of support for the trial on the part of some key personnel and groups contributed to avoidance of communications. Alternatively, triangulation, or over-involvement of the TC and lead investigator, occurred during disputes or problems occurring between groups, rather than initial problem resolution occurring between liaisons

## Discussion

We summarise six specific lessons learned from this trial as follows:

1. Clearly identified tasks and task flow maps are the first organisational priority. This involves identifying and prioritising those variables needed to satisfy the specific aims of the trial. Multiple task flow maps [36, 37] can then be generated to identify key data providers, service groups, and major data collection points. This will simplify identification of personnel and service groups to be included in the trial
2. Familiarise all personnel early with ICS structure and chains of command. Establishing clear ICS chains of command can streamline operations by facilitating coordination and scheduling of personnel, organising tasks, and identifying and correcting operational bottlenecks. However, the system can only work if trial personnel understand the ICS architecture and the lines of authority outlined in the ICS tree [23]. Clinicians may be unaccustomed to ICS systems and will have to be educated in the basic principles
3. There is no such thing as too much planning. During the early phase of this trial, pressure to get the trial launched resulted in inadequate planning. After trial launch, various operational gaps and problems could only be identified only once SOPs were put into practice. Consequently, planning was an on-going and iterative process
4. Responsibility for regulatory administration of a large trial should never rely on a single person. Early in the PUPTH trial, regulatory compliance and documentation was the responsibility of a single person, as was common at this institution. The processes involved were extremely complicated and almost incomprehensible to the lead investigator and planning committee. Therefore, it was extremely difficult to detect errors until late in the trial timeline. Subsequently, two additional expert staffers were required to examine the mountains of regulatory paperwork for due diligence, and correct mistakes and omissions in previous regulatory submissions. In large trials, additional expert team members may be required to review, cross-check, and identify and eliminate errors and omissions in a timely manner
5. Good communication is essential. The free flow of information is essential to trial success. The lead investigator and primary trial personnel must establish an atmosphere where clear expectations are identified, and personnel respect the time, expertise and effort of other trial personnel. Informal methods of communication, such as phone calls, or email are important, but frequent face-to-face meetings (with definable and actionable goals) are key to maintaining strong working relationships
6. Formally assess stakeholder relevance. A more structured and early assessment of stakeholders can improve identification and prioritisation of group members, especially when criteria are blurred. For example, in the PUPTH trial it was assumed that EMS agency medical directors could serve as EMS proxies, without the presence of representatives from the agencies themselves. However, the scope of medical director oversight refers only to patient care activities and provider practice [43]. As planning progressed, it became clear that EMS representation was essential for understanding specific agency capabilities and limitations, identifying logistic bottlenecks, finding acceptable compromises between trial demands and normal work and resource requirements, and gaining EMS provider commitment to the research plan.

Unfortunately, funding for support personnel may be limited. Clinician salary support can account for a substantial portion of available funds, leaving insufficient money for essential non-clinician support. Alternatively, the terms of some grant awards may prohibit salary support for certain staff, such as supernumerary finance or regulatory officers, thus greatly reducing operational capability. Early identification of essential support personnel and hiring constraints is required for adequate planning.

## Conclusions

The diversity and scope of tasks and responsibilities required for a large clinical trial are impossible for any one person, or even a small team, to manage with skill. Trial management is a distributed process; both broad representation and coordination of key groups and stakeholders are essential for efficient and successful trial implementation. The ICS, commonly employed in emergency management organisation, represents an innovative approach to the management of complex large clinical trials. Incorporation of ICS principles is an effective means of improving trial coordination and capability, as it is flexible, structured, and highly reliable under complex and unstable operational situations [23]. We found that application of ICS to the management of this specific clinical trial was instrumental in overcoming early stage inertia and organisational confusion. ICS allowed more rapid targeting of specific areas of trial dysfunction and bottlenecks, and identification of appropriate personnel required for problem solution, without overwhelming the lead investigator and TC. Role flexibility and role switching allowed nimble responses of the planning team and steering committee to situational changes.

However, implementation of ICS is not enough in itself to guarantee trial success. Causes of trial failure can originate at any or all organisational levels, either through overall misdirection from the top, substandard work at the implementation levels, or through omission or insufficient attention to one or more critical components of the trial. Efficient and successful implementation requires consideration of the clinical culture, and the psychological and social factors operating outside of ICS. Personnel unused to working within an ICS and who sidestep organisational procedures constitute the largest barrier to successful implementation.

## Abbreviations

CCRC: Certified Clinical Research Coordinators
DSMB: Data Safety Monitoring Board
EMS: Emergency medical services
FDA: U.S. Food and Drug Administration
GCP: Good Clinical Practices guidelines
HiPPO: Highest Paid Person’s Opinion
ICS: Incident Command System
IT: Information technology
PI: Principal investigator
POETE: Planning, Organisation, Equipment, Training, Evaluation
PR: Public relations
PUPTH: Prehospital Use Of Plasma in Traumatic Haemorrhage
SOP: Standard operating procedure
TC: Trial coordinator

## Footnotes

1 The HiPPO acronym (coined by web analytics guru Avinash Kaushik) describes the tendency for decisions to be made on the basis of ‘gut feelings’ or deference of subordinate to more senior personnel.
